# Effect of Nb^3+^ Substitution on the Structural, Magnetic, and Optical Properties of Co_0.5_Ni_0.5_Fe_2_O_4_ Nanoparticles

**DOI:** 10.3390/nano9030430

**Published:** 2019-03-13

**Authors:** Munirah. A. Almessiere, Yassine Slimani, Murat Sertkol, Muhammed Nawaz, Ali Sadaqat, Abdulhadi Baykal, Ismail Ercan, Bekir Ozçelik

**Affiliations:** 1 Department of Biophysics, Institute for Research and Medical Consultations (IRMC), Imam Abdulrahman Bin Faisal University, P.O. Box 1982, Dammam 31441, Saudi Arabia; malmessiere@iau.edu.sa (M.A.A.); yaslimani@iau.edu.sa (Y.S.); iercan@iau.edu.sa (I.E.); 2 Department of Physics, College of Science, Imam Abdulrahman Bin Faisal University, P.O. Box 1982, Dammam 31441, Saudi Arabia; 3 Deanship of Preparatory Year, Imam Abdulrahman Bin Faisal University, P.O. Box 1982, Dammam 31441, Saudi Arabia; msertkol@iau.edu.sa; 4 Department of Nanomedicine Research, Institute for Research and Medical Consultations (IRMC), Imam Abdulrahman Bin Faisal University, P.O. Box 1982, Dammam 31441, Saudi Arabia; mnnmuhammad@iau.edu.sa; 5 Mechanical and Energy Engineering Department, College of Engineering, Imam Abdulrahman Bin Faisal University, P.O. Box 1982, Dammam 31441, Saudi Arabia; sadali@iau.edu.sa; 6 Department of Physics, Faculty of Science and Letters, Cukurova University, Balcali-Adana 01330, Turkey; ozcelik@cu.edu.tr

**Keywords:** spinel ferrites, Nb substitution, low temperature magnetization, optical properties, TEM analysis

## Abstract

Co_0.5_Ni_0.5_Nb_x_Fe_2−x_O_4_ (0.00 ≤ x ≤ 0.10) nanoparticles (NPs) were prepared using the hydrothermal approach. The X-ray powder diffraction (XRD) pattern confirmed the formation of single-phase spinel ferrite. The crystallite size was found to range from 18 to 26 nm. The lattice parameters were found to increase with greater Niobium ion (Nb^3+^) concentration, caused by the variance in the ionic radii between the Nb^3+^ and Fe^3+^. Fourier transform infrared analysis also proved the existence of the spinal ferrite phase. The percent diffuse reflectance (%DR) analysis showed that the value of the band gap increased with growing Nb^3+^ content. Scanning electron microscopy and transmission electron microscopy revealed the cubic morphology. The magnetization analyses at both room (300 K, RT) and low (10 K) temperatures exhibited their ferromagnetic nature. The results showed that the Nb^3+^ substitution affected the magnetization data. We found that Saturation magnetization (*M_s_*), Remanence (*M_r_*), and the Magnetic moment ($n_{B}$) decreased with increasing Nb^3+^. The squareness ratio (SQR) values at RT were found to be smaller than 0.5, which postulate a single domain nature with uniaxial anisotropy for all produced ferrites. However, different samples exhibited SQRs within 0.70 to 0.85 at 10 K, which suggests a magnetic multi-domain with cubic anisotropy at a low temperature. The obtained magnetic results were investigated in detail in relation to the structural and microstructural properties.

## 1. Introduction

Spinel ferrite nanoparticle materials are highly preferred in engineering and technology applications like biomedicine, pharmaceuticals, sensors, magnetic resonance imaging, drug delivery, microwaves, high-frequency devices, information storage, and electronic chips [1,2,3,4]. The structure and electromagnetic properties of nano-spinel ferrites can be modified by the substitution of different cations. Rare earth substitutions are highly valuable for reducing the particle size and intensification of the lattice parameter [5]. In this respect, substituting rare earth (RE) cations into the spinel ferrite structure plays an important role in enhancing the dielectric, magnetic, and electric properties due to the Fe–Fe interactions caused by the spin coupling effect of 3d electrons [6]. Therefore, electrical and magnetic behavior may change when rare earth and iron interactions (3d–4f coupling) of the spinel ferrites occur. Different RE substitutions have been proven to have different results on the ferrite structure [7,8,9]. Many researchers have investigated the effects of RE substitution into cobalt spinel ferrite (CoFe_2_O_4_) in bulk form, thin film, and nanoparticles [10,11,12]. Coercivity, anisotropic constant, and uniaxial anisotropy were described as decreasing with the addition of m and Ce ions into nano-cobalt ferrites [13]. Several methods have been used to synthesize nickel-substituted cobalt ferrites, Co_1−x_Ni_x_Fe_2_O_4_, such as the auto-combustion method, aerosol route, and co-precipitation method [14,15,16]. Chen et al. [17] prepared Ni_1−x_Co_x_Fe_2_O_4_ nanoparticles (NPs) using the hydrothermal method and studied the increasing trend in saturation magnetization with increasing cobalt content, which occurred due to the substitution of an Ni^2+^ ion to a Co^2+^ ion. Maz et al. [18] synthesized Co_1−x_Ni_x_Fe_2_O_4_ nanoparticles in a chemical co-precipitation process and noticed the increasing trend of coercivity (H_c_) and saturation magnetization (*M_s_*) with increasing cobalt content.

Coercivity (H_c_) and saturation magnetization (*M_s_*) values were shown to decrease with an increase in the Ni content in Co_1−x_Mn_x_Fe_2_O_4_ (0.0 ≤ x ≤ 0.4) nanoparticles [19]. A few papers have been published on Nd^3+^ ion-substituted cobalt-ferrite in the Fe sites. Almessiere et al. [20] reported the effect of the magnetocrystalline anisotropy constant decreasing with the addition of Nd^3+^ ions in cobalt spinel nano-ferrites Co_1−2x_Ni_x_Mn_x_Fe_2−y_Nd_y_O_4_ (0.0 ≤ x = y ≤ 0.3) synthesized using the sol-gel method. Yadav et al. [21] examined the magnetic and structural properties by incorporating Nd^3+^ ions in cobalt spinel ferrite nanoparticles, CoFe_2−x_Nd_x_O_4_ (x ≤ 0.1), synthesized using the sol-gel method. Tahar et al. [22] investigated the RE = La, Ho, Tb, Ce, Gd, Nd, and Sm substitutions in CoFe_1.9_RE_0.1_O_4_ nanoparticles. Aside from these RE substitutions, Nd^3+^ ions considerably decrease the coercivity and saturation magnetization. Zhao et al. [23] reported that coercivity and saturation magnetization slightly increased with Nd^3+^ substitution in cobalt ferrite nanocrystals CoFe_2−x_Nd_x_O_4_ synthesized by the emulsion method. In this study, the effect of Nb^3+^ substituted in the Co-Ni spinel ferrite on the structural, optical, and magnetic properties are discussed in detail.

## 2. Experimental Materials and Methods

### 2.1. Materials and Instruments

A Rigaku Benchtop Miniflex X-ray diffraction (XRD) diffractometer (Tokyo, Japan) with Cu K_α_ radiation at room temperature (RT) over the 2θ range from 20° to 70° was used for the structural analysis. Scanning electron microscopy (SEM, FEI Titan ST, Hillsboro, OR, USA) along with energy dispersive X-ray spectroscopy (EDX) and transmission electron microscopy (TEM; FEI, Morgagni 268, Prague, Czech Republic) were used for the morphological and composition analyses. Spectral analysis of all products was performed via Fourier transform infrared (FT-IR) spectroscopy (Bruker, Berlin, Germany). Ultraviolet-visible (UV-vis) diffuse reflectance (DR%, Shimadzu, Tokyo, Japan) spectra were recorded in the 200 to 800 nm wavelength range using a DR spectrophotometer. The magnetic properties of the products were measured using a Quantum Design SQUID-PPMS vibrating sample magnetometer (PPMS DynaCool, Quantum Design, San Diego, CA, USA).

Cobalt (II) nitrate (Co(NO_3_)_2_), nickel (II) nitrate (Ni(NO_3_)_2_), iron (III) nitrate hexahydrate (Fe(NO_3_)_3_.6H_2_O), and niobium (V) nitrate (Nb(NO_3_)_5_) were received from Sigma-Aldrich (St. Louis, MO, USA) and used as received.

### 2.2. Procedure

The hydrothermal method approach was used to synthesize Co_0.5_Ni_0.5_Nb_x_Fe_2−x_O_4_ (0.00 ≤ x ≤ 0.10) nanoparticles. Stoichiometric amounts of iron, nickel, and cobalt nitrates were dissolved in 50 mL deionized (DI) H_2_O. Niobium nitrate was dissolved in concentrated HCl in a separate beaker with vigorous stirring. Then, the two solutions were mixed together and stirred for an extra 30 min and the pH of the resulting solution was adjusted to 11 by adding sodium hydroxide (NaOH) with continuous stirring for 30 min. Before transfer to a Teflon-lined vessel, the mixture was pretreated in an ultrasonic water bath for 30 to 40 min. The final solution was transferred to a stainless-steel Teflon autoclave (200 mL) and placed in an oven at 180 °C for 10 h. The resulting product was washed with hot deionized water three times and left to dry overnight at 80 °C for 5 h.

## 3. Results and Discussion

### 3.1. XRD Analysis

**Figure 1:**
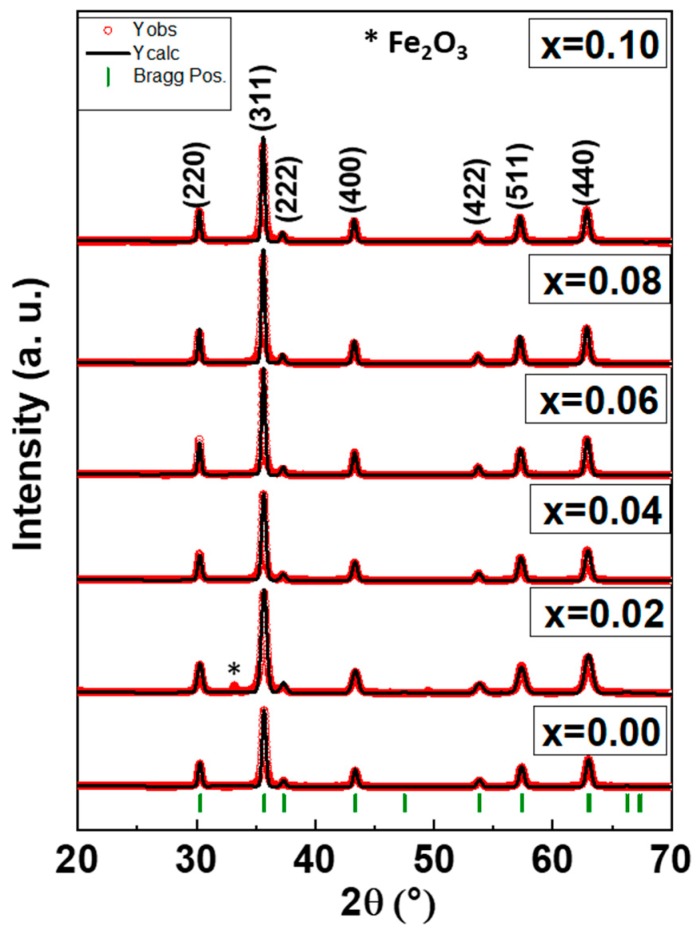
X-ray diffraction (XRD) powder patterns of the Co_0.5_Ni_0.5_Nb_x_Fe_2−x_O_4_ (0.0 ≤ x ≤ 1.0) nanoparticles.

Figure 1 displays the crystal structure of the Co_0.5_Ni_0.5_Nb_x_Fe_2−x_O_4_ (0.00 ≤ x ≤ 0.10) nanoparticles. The XRD analysis showed the signature peaks of a single phase of Co spinel ferrite with no presence of any extra secondary phase, which means that the Nb^3+^ was well dissolved in the Co–Ni crystal. Only the x = 0.02 sample had a minor amount of the Fe_2_O_3_ phase as an impurity. The structural parameters were calculated through Rietveld refinement using the XRD experimental data, as registered in Table 1. We found that the lattice parameters increased with the rise in the Nb^3+^ amount due to the larger ionic radius of Nb^3+^ (0.72 Å) in comparison with Fe^3+^ (0.64 Å), which caused stress in the lattice. The crystallite sizes were estimated to range from 18 to 26 nm.

### 3.2. Spectral Analysis

**Figure 2:**
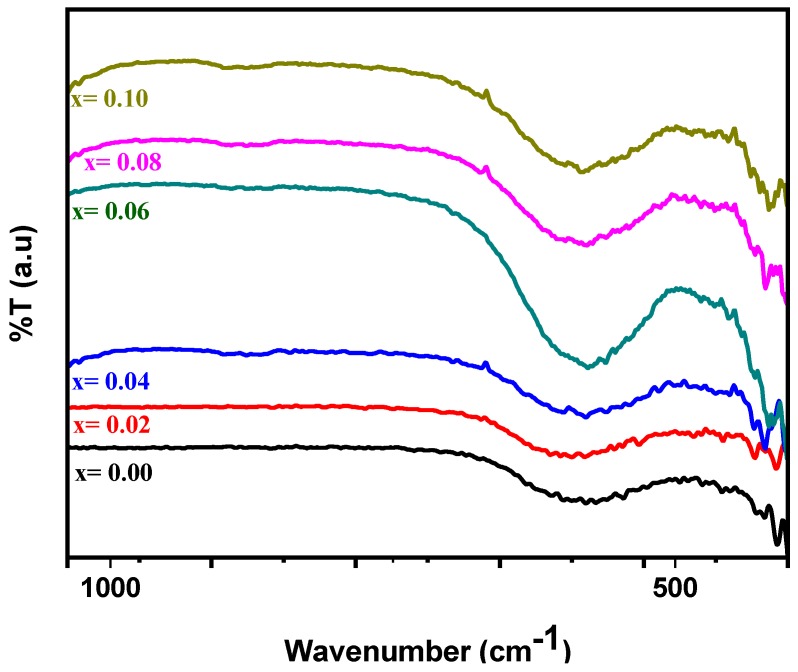
Fourier transform infrared (FT-IR) spectra of the Co_0.5_Ni_0.5_Nb_x_Fe_2−x_O_4_ (0.0 ≤ x ≤ 1.0) nanoparticles.

Figure 2 highlights the FT-IR spectra of Co_0.5_Ni_0.5_Nb_x_Fe_2−x_O_4_ (0.00 ≤ x ≤ 0.10). The strongest stretching bands at 584.36 and 410.7 cm^−1^ represent the Co spinel ferrite structure. These bands can be attributed to the stretching of the vibration band between F and O. The vibration bands shifted toward higher wavenumbers when the Nb^3+^ content increased, which was due to the variation in the bond length as a result of the larger ionic radii of Nb^3+^ [24,25,26].

### 3.3. Morphological Analysis

**Figure 3:**
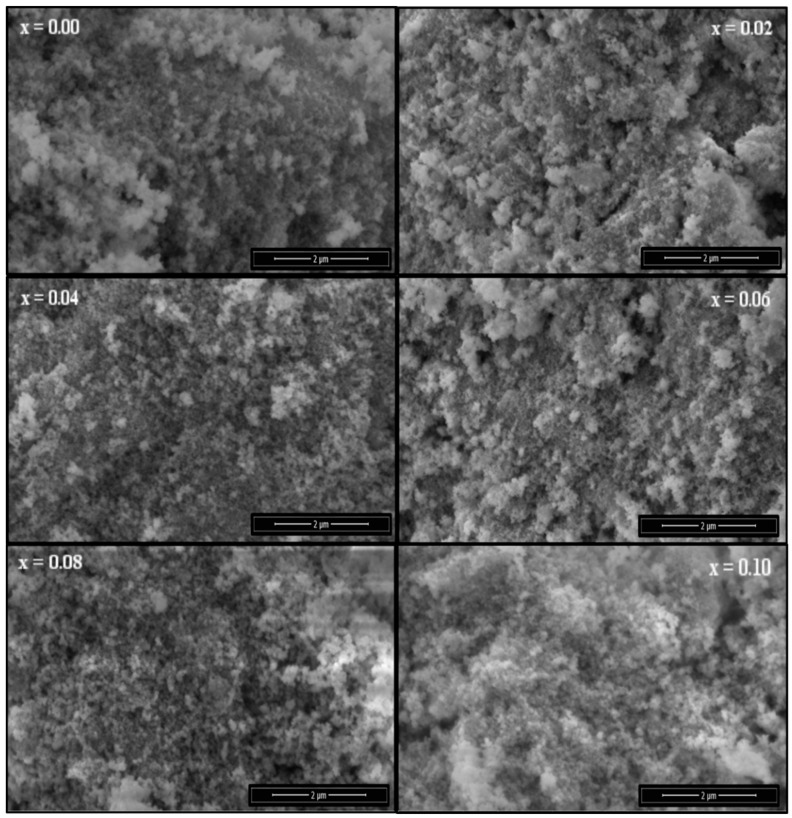
Scanning electron microscope (SEM) micrographs of the Co_0.5_Ni_0.5_Nb_x_Fe_2−x_O_4_ (0.0 ≤ x ≤ 0.10) nanoparticles.

**Figure 4:**
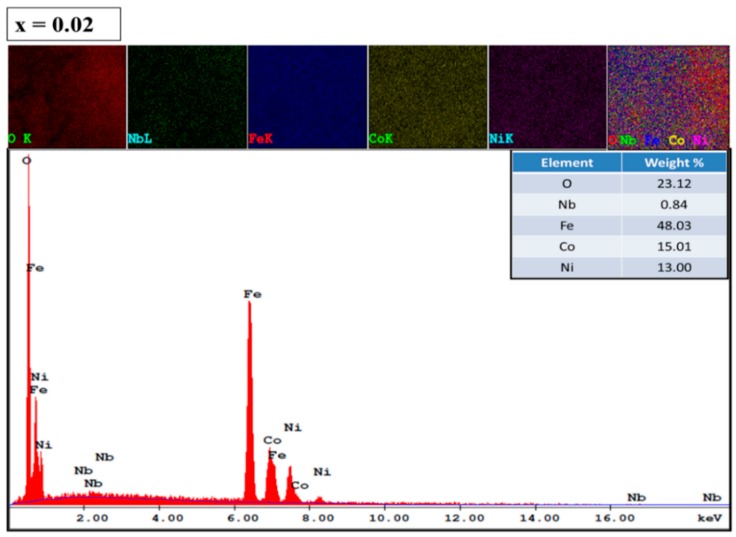

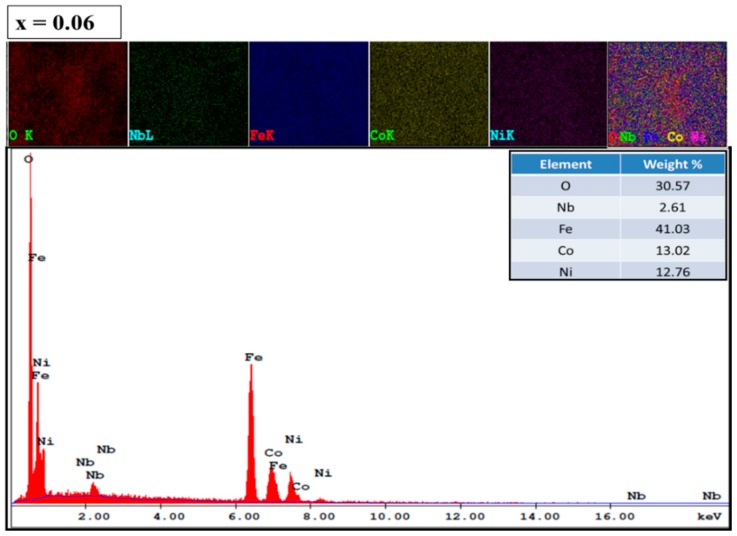
Energy dispersive X-ray (EDX) spectrographs and elemental mapping images of the Co_0.5_Ni_0.5_Nb_x_Fe_2−x_O_4_ nanoparticles for x = 0.02 and 0.06.

**Figure 5:**
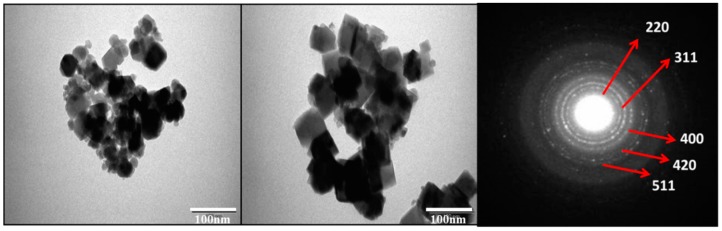
Transmission electron diffraction (TEM) images (with different magnetization) and selected area electron diffraction (SAED) pattern of Co_0.5_Ni_0.5_Nb_x_Fe_2−x_O_4_ for x = 0.06 nanoparticles.

The Field emission scanning electron microscope (FE-SEM) microstructure of the Co_0.5_Ni_0.5_Nb_x_Fe_2−x_O_4_ (0.00 ≤ x ≤ 0.10) nanoparticles is depicted in Figure 3. The images exhibited a higher agglomeration cubic shape with an average grain size of less than 27 nm, which agreed with the crystal size estimated by XRD. The EDX spectrum and elemental mapping confirmed the existence of Co, Ni, Nb, and O in compositions x = 0.02 and 0.06, as shown in Figure 4. The quantitative analysis of selected samples that recorded the atomic weigh of x = 0.02 and 0.06 showed that the estimated values were close to the expected values for the samples. The TEM and SAED (Selected area electron diffraction) pattern of the Co_0.5_Ni_0.5_Nb_x_Fe_2−x_O_4_ (x = 0.06) nanoparticles are shown in Figure 5. The images confirmed the cubic spinel structure and aggregate.

### 3.4. Optical Analysis

**Figure 6:**
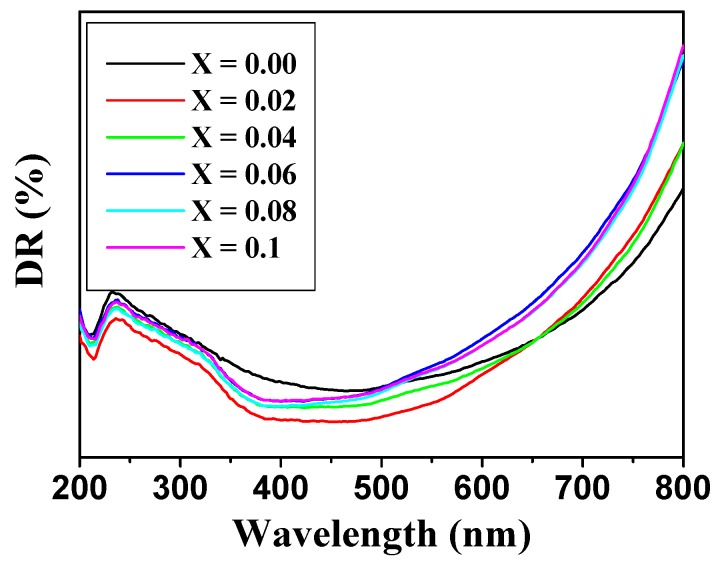
DR% spectra of the Co_0.5_Ni_0.5_Nb_x_Fe_2−x_O_4_ (0.0 ≤ x ≤ 0.10) nanoparticles.

**Figure 7:**
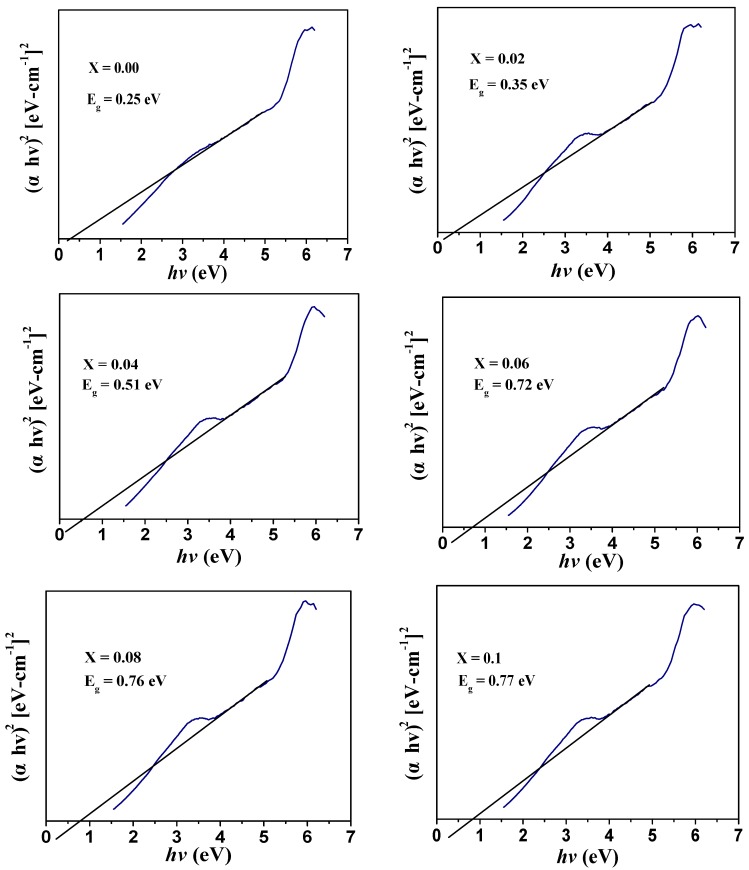
[αhv]^2^ versus *hv* graphs of the Co_0.5_Ni_0.5_Nb_x_Fe_2−x_O_4_ (0.0 ≤ x ≤ 0.10) nanoparticles.

The optical properties of Co_0.5_Ni_0.5_Nb_x_Fe_2−x_O_4_ (0.00 ≤ x ≤ 0.10) nanoparticles were studied using a DR-UV-visible spectrophotometer ranging from 200 to 800 nm. Figure 6 shows that the compositions at different concentrations exhibited spectra in the visible range. The Kubelka–Munk model was used to compute the optical band gap energy (*E_g_*) [27]. The band gap energy was calculated using a plot of (α*hv*)^2^ vs. photon energy (*hv*), which is also called the Tauc plot, of the Co_0.5_Ni_0.5_Nb_x_Fe_2−x_O_4_ (0.00 ≤ x ≤ 0.10) nanoparticles (Figure 7).

The band gap values were 0.25, 0.35, 0.51, 0.72, 0.76, and 0.77 eV for x = 0.00, 0.02, 0.04, 0.06, 0.08, and 0.1, respectively. When the value of x increased, the band gap value increased. The increase in the band gap value was ascribed to the development of the energy level or interface defects in M. Almessiere et al. [28]. The increase in the band gap could also be due to the synergistic effect of nano-ferrite with niobium, which decreases the electron hole recombination [29].

### 3.5. Magnetization Investigations

**Figure 8:**
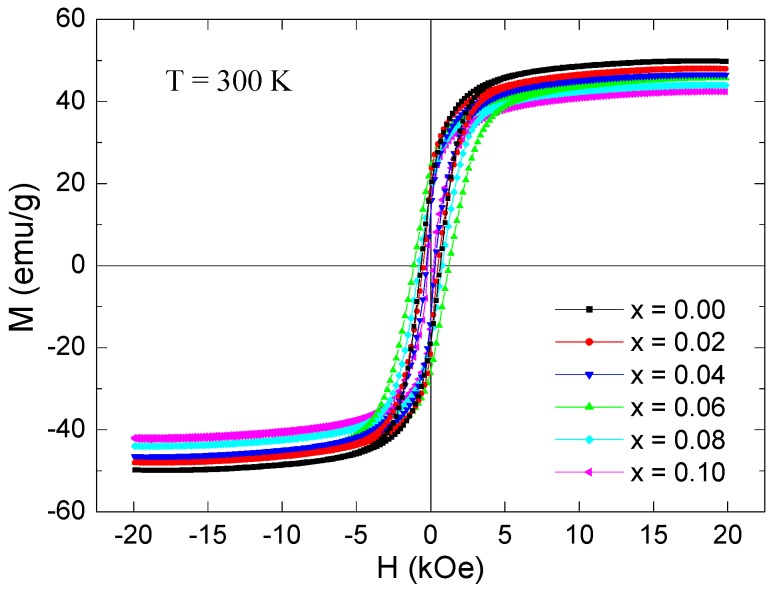
Magnetization versus applied field of ±20 kOe, M(H), for the Co_0.5_Ni_0.5_Fe_2−x_O_4_ (0.0 ≤ x ≤ 0.10) nanoparticles at RT.

**Figure 9:**
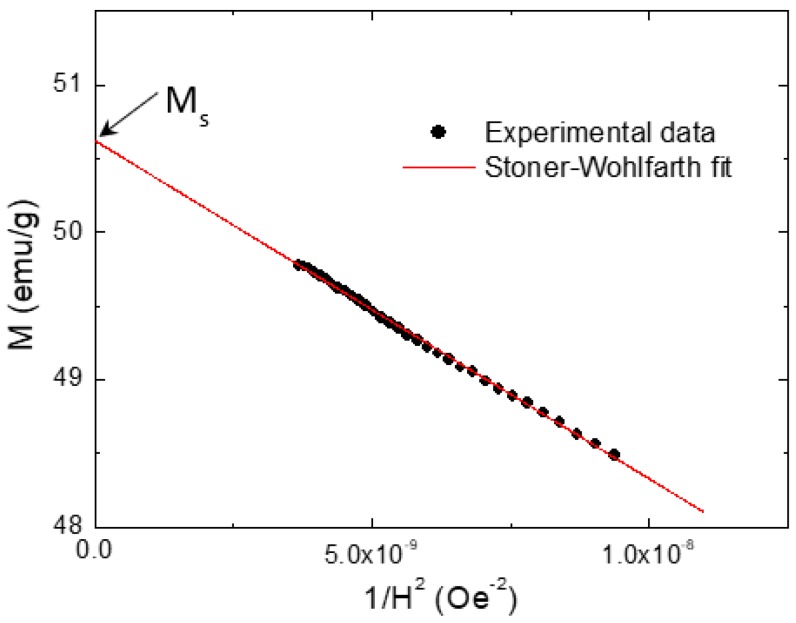
A schematic example of the M vs. 1/H^2^ plot for the Co_0.5_Ni_0.5_Fe_2_O_4_ nanoparticles at RT.

The magnetization plots against an applied magnetic field of ±20 kOe, M(H) for all the Co_0.5_Ni_0.5_Nb_x_Fe_2−x_O_4_ (0.00 ≤ x ≤ 0.10) nanoparticles performed at RT are illustrated in Figure 8. Table 2 summarizes the various deduced magnetic parameters for all the Co_0.5_Ni_0.5_Nb_x_Fe_2−x_O_4_ (0.00 ≤ x ≤ 0.10) nanoparticles at RT. The Nb^3+^ substitution in the Fe^3+^ sites altered the magnetic properties of the CoNi ferrite. The different CoNi ferrites displayed remanence magnetization (*M_r_*) ranging from 13.00 to 23.66 emu/g. *H_c_* ranged from 207.31 to 1129.92 Oe. The *M_max_*_,20_ (magnetization at a maximum field of 20 kOe) was found to be between 42.36 and 49.77 emu/g. The Stoner–Wohlfarth (S–W) theory was used to extract the saturation magnetization (*M_s_*) [30,31,32]. An example of the estimation of *M_s_* for the x = 0.00 sample is shown in Figure 9. The extrapolation of this plot at high magnetic fields approaching zero produces the *M_s_* value. The *M_s_* values of the Co_0.5_Ni_0.5_Nb_x_Fe_2−x_O_4_ (0.00 ≤ x ≤ 0.10) nanoparticles ranged from 43.15 to 50.62 emu/g at RT. According to the obtained findings, we confirmed that the differently produced Co_0.5_Ni_0.5_Nb_x_Fe_2−x_O_4_ (0.00 ≤ x ≤ 0.10) nanoparticles have a soft ferromagnetic (FM) nature at RT.

**Figure 10:**
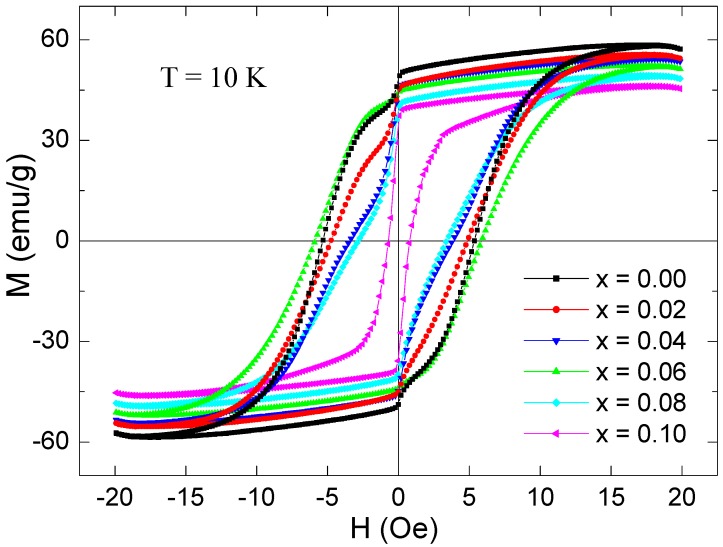
Magnetization against an applied field of ±20 kOe, M (H), for all of the Co_0.5_Ni_0.5_Fe_2−x_O_4_ (0.0 ≤ x ≤ 0.10) nanoparticles at 10 K.

The M(H) were also performed for all of the Co_0.5_Ni_0.5_Nb_x_Fe_2−x_O_4_ (0.00 ≤ x ≤ 0.10) nanoparticles at 10 K (Figure 10). The deduced magnetic parameters at 10 K are listed in Table 3. *H_c_* ranged from 708.93 to 5882.24 Oe. *M_r_* ranged from 34.11 to 48.09 emu/g. The *M_max_*_,20_ values ranged from 45.36 to 57.22 emu/g. *M_s_* varied from 45.71 to 57.96 emu/g. Spinel ferrite nanoparticles have been reported to have a superparamagnetic threshold below 10 nm [33]. Since our nanoparticles had dimensions larger than 10 nm, the effect of the superparamagnetic state nanoparticles on lower magnetization was neglected. The obtained magnetic results at 10 K revealed the semi-hard FM nature of all products. Compared to 300 K, the *M_s_*, *M_r_*, and *H_c_* showed a remarkable increase at 10 K. This increase was due to reduced thermal fluctuations of the magnetic moments [34,35]. In the literature, the anisotropy contribution of RE ions has been reported via spin-orbit coupling when they occupy the B sites of spinel ferrites [31,36]. This is one of the reasons for the observation of higher coercivities at CoMn ferrites doped with rare-earth ions.

At both measurement temperatures, the x = 0.00 sample had the highest magnitudes of *M_s_*, which were about 49.77 and 57.22 emu/g at 300 and 10 K, respectively. Likewise, the x = 0.00 product exhibited the maximum *M_r_* values with magnitudes of 18.47 and 48.09 emu/g at 300 and 10 K, respectively. The magnetization magnitudes found in the present study for the non-substituted sample were comparable to those of the CoFe_2_O_4_ and NiFe_2_O_4_ inverse spinel ferrites [37,38], but they are larger than those reported in the literature for Co_0.5_Ni_0.5_Fe_2_O_4_ [39]. The obtained magnitudes were smaller than those reported for both the bulk CoFe_2_O_4_ and NiFe_2_O_4_ inverse spinel ferrites [33,40]. The lower *M_s_* and *M_r_* magnitudes, in comparison to that of the bulk samples, were largely attributed to the smaller crystallite size, which leads to a structural disorder on the surface since the spin disorder will be significant when the volume and surface ratio are important [41]. Spin canting as a result of antiferromagnetic interaction competition, the construction of a magnetic inactive layer, the non-collinear arrangement of the magnetic moments of Fe^3+^ ions, and the disordered cations distributions on the surface could all explain the lowered magnetization magnitudes [42,43].

At both temperatures, the highest *H_c_* was observed for x = 0.06 and the lowest for x = 0.10. Various parameters governed the coercivity, like grain size, magnetic particle morphology, magnetocrystalline anisotropy, strains and exchange coupling between the collinear spins in the core, and the canted spins on the surface [34,35]. The improvement in coercive field can be principally attributed to the increase of magnetocrystalline anisotropy [44,45]. Equation (1) describes the proportionality between the coercivity *H_c_* and magnetic anisotropy constant *K_a_* [44].

(1) $$H_{c}\propto \frac{2K_{a}}{\mu_{o}M_{s}}$$

where $\mu_{o}$ is the permeability constant. When the magnetic anisotropy increases with increasing substitution content, coercivity grows. To determine the anisotropy constant *K_a_*, the expression used to estimate the values of *M_s_* according to the S–W fit is shown below [31,32].

(2) $$M=M_{s}\left(1-\frac{\beta }{H^{2}}\right)$$

Consequently, the slope of the linear fitting provides the constant *β*, which is related to the magnetocrystalline anisotropy constant *K_a_*. Once the values of the *β* constant are determined, the magnetic anisotropy constant (*K_a_*) can be estimated by using the equation below [31,32].

(3) Ka=Ms(15 β4)12

The deduced *K_a_* values at 300 and 10 K are listed in Table 2 and Table 3, respectively. The magnetocrystalline anisotropy was the maximum for the x = 0.06 product and was the minimum for x = 0.10. This result explains the highest coercivity in the x = 0.06 sample.

The Nb^3+^ substitution led to a gradual reduction in the *M_s_* and *M_r_* values. The lowest magnitudes were observed for the Co_0.5_Ni_0.5_Fe_1.9_Nb_0.1_O_4_ (x = 0.1) sample. The minimum *M_s_* values belonging to the x = 0.1 product were about 43.15 and 45.71 emu/g at 300 and 10 K, respectively. The minimum *M_r_* for x = 0.1 were 13.00 and 34.11 emu/g at 300 and 10 K, respectively. The evolutions in the *M_r_* values showed a similar trend to *M_s_* with respect to the Nb^3+^ concentration. It has been reported previously that evolutions in the *M_r_* values depend principally on evolutions in *M_s_* and on the net alignment of magnetization grains derived from super-exchange interactions between the magnetic particles [32]. 

Numerous factors can affect the magnetic properties of spinel ferrites, including the crystallite size change, variations in magnetic moments ($n_{B}$), variations in the nature and concentration of different sites, and the preferred site occupancy of different ions [36]. The local strains and the super-exchange interactions between different ions might influence the magnetic parameters [34,35]. Principally, the magnetic moment of spinel ferrites is derived from the iron ions and their distribution in the crystal sites. The A–A and B–B interactions were unimportant. However, the A–B exchange interactions were dominant. Consequently, any factors that affect the strength of various exchange interactions will modify the magnetization. In our case, the observed decrease in *M_s_* and *M_r_* values with Nb^3+^ substitution was attributable to the weakening of the exchange interactions in the Fe sites. The ions of the host Fe^3+^ (0.62 Å) displayed a slightly smaller ionic radius when compared to that of Nb^3+^ (0.72 Å). The contrast of magnetic moments and ionic radii of the host and substituted ions might produce a non-collinear ferromagnetic arrangement and local strains that cause the disorder and variations in electronic states in the hexaferrite systems [32,34,35]. The substitution of Fe^3+^ ions with Nb^3+^ ions resulted in increasing the distance separating the magnetic ions and, therefore, decreasing the strength of the A–B super-exchange interactions. The relation between the magnetic moment $\left(n_{B}\right)$ and *M_s_* is given by the formula below [36].

$$n_{B}=\frac{Molecular\;Weight\times M_{s}}{5585}$$

The estimated $n_{B}$ values of all of the Co_0.5_Ni_0.5_Nb_x_Fe_2−x_O_4_ (0.00 ≤ x ≤ 0.10) nanoparticles at 300 and 10 K are summarized in Table 2 and Table 3, respectively. The decrease in $n_{B}$ values resulting from the weakening of the super-exchange interactions among the various sites led to a decrease in the $n_{B}$ values. In our case, $n_{B}$ was found to decrease with increasing Nb^3+^ content. The x = 0.00 sample where the *M_s_* value was the highest displayed the greatest $n_{B}$. The x = 0.10 sample where the *M_s_* value was the lowest displayed the lowest $n_{B}$. This indicates a weakening of the super-exchange interactions.

The squareness ratios (*SQR* = *M_r_*/*M_s_*) were calculated for all the Co_0.5_Ni_0.5_Nb_x_Fe_2−x_O_4_ (0.00 ≤ x ≤ 0.10) nanoparticles at 300 and 10 K. According to the S–W theory, the SQR can take two values including one around 0.83 associated with the cubic anisotropy, and another around 0.5 that corresponds to uniaxial anisotropy [30,32]. The findings of *SQR* equal to or above 0.5 indicated that the particles were in the single magnetic domain, and those below 0.5 could be attributed to the formation of a multi-domain structure [46]. As can be seen from the tables, the *SQR* at RT was found to be around 0.509 for the x = 0.06 sample, which suggests a single magnetic domain with uniaxial anisotropy. However, the other samples displayed *SQRs* that ranged from 0.31 to 0.42, which were less than 0.50 and can be attributed to surface spin disorder effects. This *SQR* was lower than 0.5, which indicates the formation of a multi-domain structure with uniaxial anisotropy. At 10 K, the different samples were found to have *SQRs* ranging between 0.70 and 0.85, which are greater than 0.5. This suggests a single magnetic domain with cubic anisotropy.

## 4. Conclusions

A series of Nd^3+^-substituted Co-Ni ferrite was synthesized via the hydrothermal approach. The XRD and FT-IR analyses proved the existence of single-phase spinel Co-ferrite. TEM analyses showed the hexagonal morphology of the products with minor agglomeration. The optical results showed that the band gap of the Co_0.5_Ni_0.5_Nb_x_Fe_2−x_O_4_ (0.00 ≤ x ≤ 0.10) nanoparticles were 0.25, 0.35, 0.51, 0.72, 0.76, and 0.77 eV, respectively. The M(H) analyses showed an FM comportment at both RT and 10 K for the Co_0.5_Ni_0.5_Nb_x_Fe_2−x_O_4_ (0.00 ≤ x ≤ 0.10) nanoparticles. The magnetic parameters strongly depend on temperature and Nb substitution content. The deduced *M_s_*, *M_r_*, and $n_{B}$ values were the highest for the x = 0.00 sample and decreased with increasing Nb substitution. This effect is due to the weakening of super-exchange interactions, the creation of local strains, the preferred site occupancy, and the decrease in the magnetic moments ($n_{B}$) with respect to the Nb content. The SQR values at RT were found to be smaller than 0.5, which postulates a single domain nature with uniaxial anisotropy for all the produced ferrites. However, the different samples exhibited SQRs in the 0.70–0.85 range at 10 K, which suggests a magnetic multi-domain with cubic anisotropy at a low temperature.

## Figures and Tables

**Table 1:** Nb content and refined structural parameters (a is the lattice parameter, V is the volume of the cell, D_XRD_ is the crystallite size obtained from the broadening of the highest peak by using the Scherrer method, *χ*^2^(chi^2^) is the squared ratio, R_Bragg_ is the Bragg factor) for the Co_0.5_Ni_0.5_Nb_x_Fe_2−x_O_4_ (0 ≤ x ≤ 1.0) nanoparticles.

x	a (Å)	V (Å)^3^	D_XRD_ (nm) ± 0.09	*χ*^2^(chi^2^)	R_Bragg_
0.00	8.345 (1)	581.15	22.86	1.75	12.67
0.02	8.345 (9)	581.33	18.71	1.32	3.12
0.04	8.352 (3)	582.67	24.18	1.52	3.99
0.06	8.356 (6)	583.57	27.59	1.32	2.48
0.08	8.362 (3)	584.76	26.86	1.77	7.88
0.10	8.362 (4)	584.77	24.70	1.32	8.77

**Table 2:** Magnetic parameters of the Co_0.5_Ni_0.5_Nb_x_Fe_2−x_O_4_ (0 ≤ x ≤ 1.0) nanoparticles at room temperature (RT) (*M_max_*_,20_: max magnetization at 20 kOe, *M_s_*: saturation magnetization, *M_r_*: remanence magnetization, *K_a_*: magnetic anisotropy constant, SQR: squareness ratio, *H_c_*: coercivity and *n_B_*: magnetic moment).

x	*M_max_*_,20_ (emu/g)	*M_s_* (emu/g)	*M_r_* (emu/g)	*K_a_* (Erg/g)	SQR	*H_c_* (Oe)	$n_{B}$ $\left(\mu_{B}\right)$
0.00	49.77	50.62	18.47	1.96 × 10^5^	0.365	648.11	2.13
0.02	47.98	48.81	20.9	1.74 × 10^5^	0.428	509.9	2.06
0.04	46.57	47.36	13	1.78 × 10^5^	0.274	286.11	2.00
0.06	45.68	46.52	23.66	2.16 × 10^5^	0.509	1129.92	1.97
0.08	43.93	44.71	18.5	1.77 × 10^5^	0.414	775.67	1.90
0.10	42.36	43.15	13.48	1.63 × 10^5^	0.312	207.31	1.84

**Table 3:** The deduced magnetic parameters of the Co_0.5_Ni_0.5_Nb_x_Fe_2__−__x_O_4_ (0 ≤ x ≤ 1.0) nanoparticles at 10 K (*M_max_*_,20_: max magnetization at 20 kOe, *Ms*: saturation magnetization, *Mr*: remanence magnetization, *Ka*: magnetic anisotropy constant, SQR: squareness ratio, *H_c_*: coercivity, and *n_B_*: magnetic moment).

x	*M_max_*_,20_ (emu/g)	*M_s_* (emu/g)	*M_r_* (emu/g)	*K_a_* (Erg/g)	SQR	*H_c_* (Oe)	$n_{B}$ $\left(\mu_{B}\right)$
0.00	57.22	57.96	48.09	4.30 × 10^5^	0.830	5335.84	2.43
0.02	54.44	55.25	44.47	3.98 × 10^5^	0.805	4760.39	2.33
0.04	53.53	54.26	44.24	3.78 × 10^5^	0.815	3440.9	2.29
0.06	51.23	52.05	44.08	5.24 × 10^5^	0.847	5882.24	2.21
0.08	48.39	48.62	34.11	3.57 × 10^5^	0.702	2848	2.07
0.10	45.36	45.71	34.94	2.67 × 10^5^	0.764	708.93	1.95
